# Epizootic Pneumonia of Bighorn Sheep following Experimental Exposure to *Mycoplasma ovipneumoniae*

**DOI:** 10.1371/journal.pone.0110039

**Published:** 2014-10-10

**Authors:** Thomas E. Besser, E. Frances Cassirer, Kathleen A. Potter, Kevin Lahmers, J. Lindsay Oaks, Sudarvili Shanthalingam, Subramaniam Srikumaran, William J. Foreyt

**Affiliations:** 1 Department of Veterinary Microbiology and Pathology, Washington State University, Pullman, Washington, United States of America; 2 Washington Animal Disease Diagnostic Laboratory, Washington State University, Pullman Washington, United States of America; 3 Idaho Department of Fish and Game, Lewiston, Idaho, United States of America; The University of Melbourne, Australia

## Abstract

**Background:**

Bronchopneumonia is a population limiting disease of bighorn sheep (*Ovis canadensis*). The cause of this disease has been a subject of debate. Leukotoxin expressing *Mannheimia haemolytica* and *Bibersteinia trehalosi* produce acute pneumonia after experimental challenge but are infrequently isolated from animals in natural outbreaks. *Mycoplasma ovipneumoniae*, epidemiologically implicated in naturally occurring outbreaks, has received little experimental evaluation as a primary agent of bighorn sheep pneumonia.

**Methodology/Principal Findings:**

In two experiments, bighorn sheep housed in multiple pens 7.6 to 12 m apart were exposed to *M. ovipneumoniae* by introduction of a single infected or challenged animal to a single pen. Respiratory disease was monitored by observation of clinical signs and confirmed by necropsy. Bacterial involvement in the pneumonic lungs was evaluated by conventional aerobic bacteriology and by culture-independent methods. In both experiments the challenge strain of *M. ovipneumoniae* was transmitted to all animals both within and between pens and all infected bighorn sheep developed bronchopneumonia. In six bighorn sheep in which the disease was allowed to run its course, three died with bronchopneumonia 34, 65, and 109 days after *M. ovipneumoniae* introduction. Diverse bacterial populations, predominantly including multiple obligate anaerobic species, were present in pneumonic lung tissues at necropsy.

**Conclusions/Significance:**

Exposure to a single *M. ovipneumoniae* infected animal resulted in transmission of infection to all bighorn sheep both within the pen and in adjacent pens, and all infected sheep developed bronchopneumonia. The epidemiologic, pathologic and microbiologic findings in these experimental animals resembled those seen in naturally occurring pneumonia outbreaks in free ranging bighorn sheep.

## Introduction

Bighorn sheep are a North American species that has failed to recover from steep declines at the turn of the 20^th^ century despite strict protections and intensive management, and two populations (Sierra Nevada and Peninsular) are currently classified as endangered [1]. Epizootic pneumonia is limiting bighorn sheep population restoration and as such, the etiology is of considerable interest. The first appearance of the disease in a population is typically in the form of epizootics that affect animals of all ages and is sometimes accompanied by high (>50%) mortality rates. Subsequently, epizootics affecting primarily lambs may occur for decades [2]. Various causes have been proposed for this disease, including lungworms (*Protostrongylus* sp.) [3]–[6], Pasteurellaceae, especially *Mannheimia (Pasteurella) haemolytica,* [7]–[12] and more recently, *Mycoplasma ovipneumoniae* [13]–[16]. In a recent comparative review of the evidence supporting each of these possible etiologies we concluded that *M. ovipneumoniae* was most strongly supported as the primary epizootic agent of bighorn sheep pneumonia [14]. However, the only two previous experimental challenge studies with *M. ovipneumoniae* either did not reproduce disease [13] or were confounded by challenges with other agents [16]. The objective of this study was to improve upon previous investigations to better assess the outcome of experimental introduction of *M. ovipneumoniae* to naïve bighorn sheep.

## Methods

### Ethics statement

This study was carried out in accordance with the recommendations in the Guide for the Care and Use of Laboratory Animals of the National Institutes of Health and in conformance with United States Department of Agriculture animal research guidelines, under protocols #03854 and #04482 approved by the Washington State University (WSU) Institutional Animal Care and Use Committee. As described in those protocols, euthanasia was performed by intravenous injection of sodium pentobarbital for animals observed to be in severe distress associated with pneumonia during the study and prior to necropsy examination for surviving animals at the end of each experiment.

### Experimental aims

Experiment 1 was conducted to investigate the transmission of *M. ovipneumoniae* to bighorn sheep and their subsequent development of disease, using an infected domestic sheep source. Experiment 2 was conducted to investigate experimental direct *M. ovipneumoniae* infection of a single bighorn sheep and the subsequent transmission of this agent to conspecifics. Both experiments were conducted in multiple pens separated by short distances, which allowed investigation of transmission to both commingled and non-commingled animals.

### Experimental animals

All experimental animals originated from herds and flocks unexposed to *M. ovipneumoniae* as determined by repeated testing with both serology on blood serum and PCR on enriched nasal swab cultures (using the methods described later in the ‘Microbiological testing’ section). In Experiment 1, three hand-reared bighorn sheep (yearling rams BHS #82 and #89 and yearling ewe BHS #07) that originated from a captive flock at WSU and three purchased domestic sheep (adult ewes DS #00 and #01 and yearling ewe DS #LA) were co-housed in three 46 m^2^ pens, with one domestic and one bighorn sheep per pen. Pens were separated by 7.6–12 m. Experiment 1 animals had all been commingled in a single pen for 104 days immediately prior to the beginning of this experiment, as previously described [15]. One of the four bighorn sheep used in that prior study had died of *M. haemolytica* pneumonia, while the other three, which had demonstrated no signs of respiratory disease in that study, were used in experiment 1. In Experiment 2, wild bighorn sheep captured from the Asotin Creek population in Hells Canyon were housed in two 700 m^2^ pens, 7.6 m apart, with three animals per pen (Pen #1: adult ewe BHS #40, yearling ewe BHS #38, and yearling ram BHS #39; Pen #2: adult ewes BHS #41 and #42 and adult ram BHS #C). The study pens had either never previously housed domestic or bighorn sheep (pen 1 in experiment 1; both pens in experiment 2) or had been rested for greater than one year since their previous occupancy by any *M. ovipneumoniae* infected sheep (pens 2 and 3 in experiment 1) prior to these experiments.

### Experimental design

#### Experiment 1

A domestic ewe (DS #00) was placed in isolation and experimentally infected with *M. ovipneumoniae.* The inoculum consisted of ceftiofur-treated (100 ug/ml, 2 hrs, 37°C; Pfizer, Florham Park, NJ) nasal wash fluids from a domestic sheep naturally colonized with *M. ovipneumoniae* [16]. Following ceftiofur treatment, no aerobic bacterial growth was observed from the nasal wash fluids cultured under conditions expected to permit growth of *M. haemolytica, B. trehalosi,* or *P. multocida* (Columbia blood agar with 5% sheep blood, 35°C, overnight, 5% CO_2_). DS #00 was then challenged with the treated nasal wash fluid by infusion of 15 ml in each nares, 10 ml orally and 5 ml into each conjunctival sac. Subsequent nasal swab samples obtained on days 1, 2, 4 and 7 post-challenge were all PCR positive for *M. ovipneumoniae* using the method described later in the ‘Microbiological testing’ section confirming that the experimental infection had been successful. On post challenge day 7, DS #00 was introduced into pen #1 with BHS #82. Following commingling, DS #00 and BHS #82 were restrained for collection of nasal swab samples on days 1, 2, 4, 7, 14, 21, 28, and subsequently at 30 day intervals until the experiment was terminated. Rectal temperatures were recorded from both sheep approximately twice each week. Sheep in pens #2 (BHS #89 and DS #01) and #3 (BHS #07 and DS #LA) were restrained for rectal temperature determination and collection of nasal swabs for microbiology at approximately monthly intervals. All pens were observed daily for clinical signs of respiratory disease. The experiment was conducted October 2009–January 2010.

#### Experiment 2

BHS #39 was inoculated with *M. ovipneumoniae* just prior to its release into pen #1 with non-inoculated BHS #38 and #40. Non-inoculated BHS #C, #41, and #42 were housed in pen #2 on the same day. The inoculum for BHS #39 was prepared as described for that used in experiment 1 but originated from a different domestic sheep source. In lieu of computation of colony forming units, which is not possible for *M. ovipneumoniae* due to inconsistent growth on plated media, viable *M. ovipneumoniae* counts in the inoculum were determined using most probable number (MPN) using a custom 3×4 format: Triplicate enrichment broth tubes were inoculated at each of four decimal dilutions (10^−2^–10^−5^) of the treated nasal wash fluid [17], incubated (72 hrs, 35C) then PCR was used to detect growth of viable *M. ovipneumoniae*. The treated fluid was determined to contain 930 MPN/ml (95% confidence interval, 230 to 3800 MPN). Two of the bighorn sheep (BHS #38 and #39) in pen 1 were recaptured by drive net on day 21 of the experiment for nasal swab sampling to detect *M. ovipneumoniae* infection; otherwise, no live animal sampling was conducted in experiment #2 to reduce the risk of traumatic injury of the wild bighorn sheep involved. The experiment was conducted December 2011–June 2012.

#### Biosecurity

In both experiments, routine biosecurity measures included: 1) the pens containing the single *M. ovipneumoniae-*challenged animals (exposed pens) were located downwind of the prevailing wind direction from the pens containing no experimentally *M. ovipneumoniae* exposed animals (clean pens), 2) order of entry rules were established so that on any single day exposed pens were routinely entered by animal care staff for feeding and cleaning only after all work in clean pens had been completed, and 3) personal protective equipment (coveralls and boots) used in exposed pens were either not reused, or were sanitized prior to use in clean pens.

#### Clinical scores

Clinical score data were determined using the following cumulative point system: observed anorexia (1), nasal discharge (1), cough (2), dyspnea (1), head shaking (1), ear paresis (1) and weakness/incoordination (1).

#### Microbiological testing

Routine diagnostic testing performed by the Washington Animal Diagnostic Laboratory (fully accredited by the American Association of Veterinary Laboratory Diagnosticians) included detection of *M. ovipneumoniae-*specific and small ruminant lentivirus-specific antibodies in serum samples using competitive enzyme-linked immunosorbent assays (cELISA) [14], [18], [19], detection of *M. ovipneumoniae* colonization by broth enrichment of nasal swabs followed by *M. ovipneumoniae-*specific PCR testing of the broths [20], [21], detection of Pasteurellaceae in pharyngeal swab samples by aerobic bacteriologic cultures, and detection of exposure to parainfluenza-3, border disease, and respiratory syncytial viruses by virus neutralization antibody assays applied to serum samples.

PCR tests specific for detection of *M. haemolytica*, *B. trehalosi*, and *P. multocida*, and *lktA* (the gene encoding the principal virulence factor of *M. haemolytica* and *B. trehalosi)* were applied to DNA extracted from pneumonic lung tissues using previously described primers (Table 1) and methods with minor modifications. All reactions were conducted individually in 20 µL volumes containing 80–300 ng of template DNA. For *M. haemolytica*, *B. trehalosi*, *lktA* and *P. multocida*, reactions contained 0.5 units of HotStar Taq DNA polymerase (Qiagen), 2 µL 10x PCR buffer (Qiagen), 4 µL Q-solution (Qiagen), 40 µM of each dNTP (Invitrogen). The *M. ovipneumoniae* reaction used QIAGEN Multiplex PCR mix. Primers were used at final concentrations of 0.2 µM (*M. haemolytica*, *B. trehalosi*, *P. multocida,* and *M. ovipneumoniae*) or 0.5 µM (leukotoxin A). Each reaction included an initial activation and denaturation step (95°C, 15 min) and a final 72°C extension step (10 min for Mhgcp-2, lktA, lktA set-1, and LM primers; 9 min for KMT primers; 5 min for Btsod and Mhgcp primers). Cycling conditions were as follows: *M. ovipneumoniae*, 30 cycles of 95°C for 30 s, 58°C for 30 s, 72°C for 30 s; *B. trehalosi* and *M. haemolytica* (Mhgcp and Btsod primers), 35 cycles of 95°C for 30 s, 55°C for 30 s, 72°C for 40 s; *P. multocida* and *lktA* (lktA primers), 30 cycles of 95°C for 60 s, 55°C for 60 s, 72°C for 60 s; *M. haemolytica* (Mhgcp-2 primers), 40 cycles of 95°C for 30 s, 54°C for 30 s, 72°C for 30 s; *lktA* (lktA set-1 primers), 40 cycles of 95°C for 30 s, 52°C for 30 s, 72°C for 40 s. Leukotoxin expression was detected in Pasteurellaceae isolates by MTT dye reduction cytotoxicity assay as described previously [22].

**Table 1:** Primers and PCR reaction targets used in these experiments.

Pathogen/Virulencegene	Target	Primer Name	Sequence (5′ → 3′)	Size (bp)	Reference
*M. haemolytica*	*gcp*	MhgcpF	AGA GGC CAA TCT GCA AAC CTC G	267	[33]
		MhgcpR	GTT CGT ATT GCC CAA CGC CG		
*M. haemolytica*	*gcp*	MhgcpF2	TGG GCA ATA CGA ACT ACT CGG G	227	[34]
		MhgcpR2	CTT TAA TCG TAT TCG CAG		
*B. trehalosi*	*sodA*	BtsodAF	GCC TGC GGA CAA ACG TGT TG	144	[33]
		BtsodAR	TTT CAA CAG AAC CAA AAT CAC GAA TG		
*P. multocida*	*kmt1*	KMT1T7	ATC CGC TAT TTA CCC AGT GG	460	[35]
		KMT1SP6	GCT GTA AAC GAA CTC GCC AC		
Pasteurellaceae leukotoxin	*lktA*	lktAF	TGT GGA TGC GTT TGA AGA AGG	1,145	[36]
		lktAR	ACT TGC TTT GAG GTG ATC CG		
*M. haemolytica* leukotoxin	*lktA*	lktAF set-1	CTT ACA TTT TAG CCC AAC GTG	497	[34]
		lktAR set-1	TAA ATT CGC AAG ATA ACG GG		
*Mycoplasma ovipneumoniae*	16s rDNA	LMF	TGA ACG GAA TAT GTT AGC TT	361	[20], [21]
		LMR	GAC TTC ATC CTG CAC TCT GT		
*Mycoplasma ovipneumoniae*	16S–23S IGS	MoIGSF	GGA ACA CCT CCT TTC TAC GG	Variable∼490	[23]
		MoIGSR	CCA AGG CAT CCA CCA AAT AC		

The 16S–23S ribosomal operon intergenic spacer (IGS) regions of *M. ovipneumoniae* recovered from animals in these studies were PCR amplified (Table 1) and sequenced as previously described [23].

#### 16S rDNA analyses to identify the predominant bacterial flora in pneumonic lung tissues

In previous studies, culture-independent evaluation of the microbial flora of lung tissues in naturally occurring bighorn sheep pneumonia revealed a polymicrobial flora late in the disease course [13], [23]. For comparison, we applied the same methods to lung tissues of the experimentally challenged animals in this study. Note that more sensitive detection of specific respiratory pathogens was provided by the PCR assays described earlier, whereas these 16S studies were designed instead to identify the numerically predominant bacteria in affected lungs. The library size used was based on the binary distribution to provide a 95% chance of detection of each taxon comprising 10% or more of the ribosomal operon frequency in the source tissue. Two 1 g samples of pneumonic lung tissues were aseptically collected from sites at least 10 cm apart, homogenized by stomaching, and DNA was extracted (DNeasy tissue kit; Qiagen, Valencia, CA) from 100 uL aliquots of each homogenate. 16S rDNA segments were PCR amplified and cloned as described [13]. Insert DNA was sequenced from 16 clones derived from each of the two homogenates from each animal, and each sequence was attributed to species (≥99% identity) or genus (≥97% identity) based on BLAST GenBank similarity [24].

## Results

### Experiment 1

**Figure 1:**
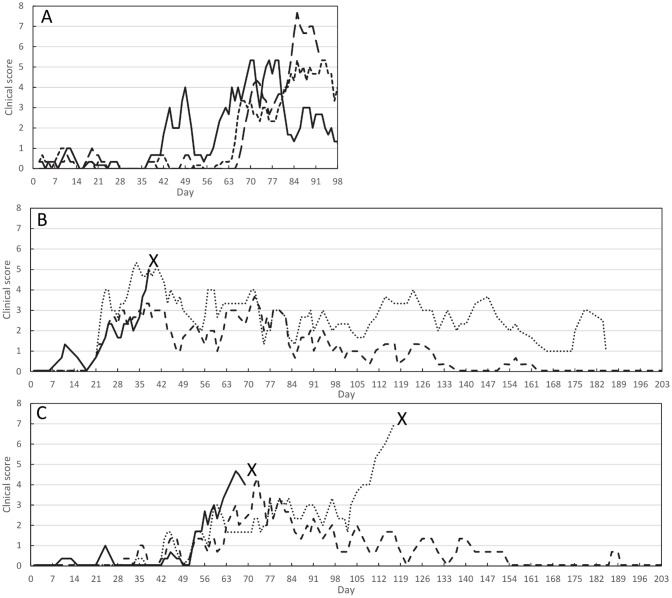
Clinical signs exhibited by *M. ovipneumoniae* infected bighorn sheep. Clinical scores (3-day moving averages) of bighorn sheep following introduction of *M. ovipneumoniae*: A) Experiment 1, 3 separate pens; solid line, Pen 1, BHS #82; dashed line, Pen 2, BHS #89; dotted line, Pen 3, BHS #07; B) Experiment 2, Pen 1: solid line, BHS #39 (died day 34); dashed line, BHS #40; dotted line; BHS #38.; C) Experiment 2, Pen 2: solid line, BHS #42 (euthanized day 109); dotted line, BHS #41 (died day 65); dashed line, BHS #C.

**Figure 2:**
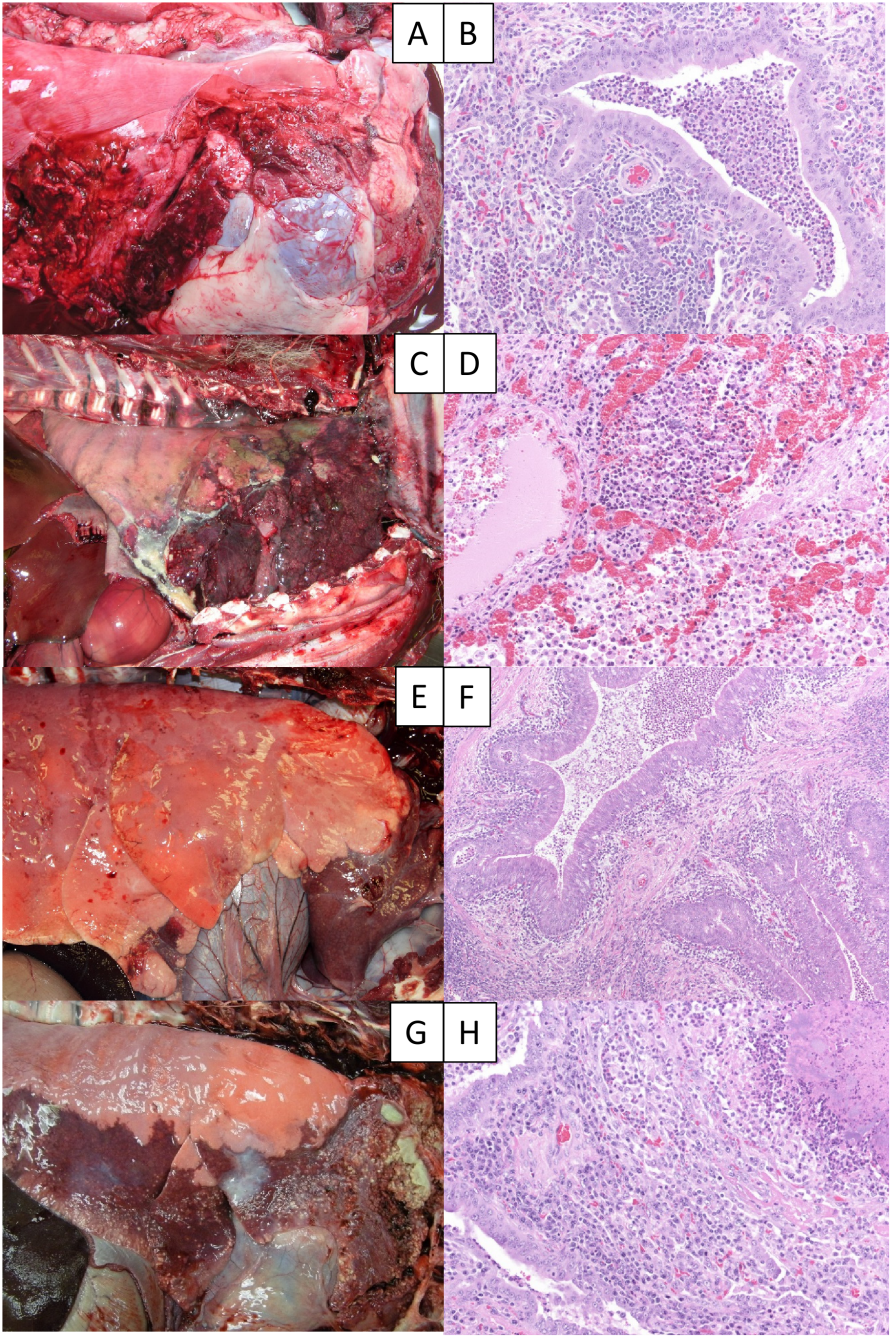
Gross and histologic lesions in lungs of bighorn sheep experimentally infected with *M. ovipneumoniae*. Images of BHS #82 (A, B), BHS #39 (C, D), BHS #C (E, F) and BHS #42 (G, H). Original magnification of histologic images was 200X (B, D, H) or 100X (F).

*M. ovipneumoniae* infection of DS #00, introduced into pen 1 to start the experiment, was confirmed by positive nasal swab samples obtained on days 1, 4, and 7 after inoculation prior to its introduction into pen #1, and on days 1, 2, 4, 7, 14, 21, 28, 60 and 90 after its introduction into pen #1, confirming that the experimental colonization had been successful and maintained throughout experiment 1. *M. ovipneumoniae* was first detected in the bighorn sheep (BHS #82) commingled with DS #00 in pen #1 on day 28, and subsequent tests on days 60 and 90 were also positive. BHS #82 developed signs of respiratory disease including nasal discharge (onset day 37); coughing and fever (onset day 42); and lethargy and ear paresis (onset day 61) (Figure 1a). Signs of respiratory disease were observed in the bighorn sheep in pens #2 (BHS #89) and #3 (BHS #07) beginning on days 62 and 67, respectively; these signs also included fever, lethargy, paroxysmal coughing, nasal discharge, head shaking, and drooping ears. No signs of respiratory disease were observed in the commingled domestic sheep at any time during the experiment. *M. ovipneumoniae* was detected in nasal swab samples from all bighorn and domestic sheep in pens #2 and #3 when sampled on day 70. The bighorn sheep were euthanized for necropsy on days 93 (BHS #89) and 99 (BHS #82 and #07). At necropsy, significant abnormal findings were limited to the respiratory tract. Bronchopneumonia affecting 25–50% of the lung volume was observed in all three bighorn sheep (Figure 2). Histopathological examination revealed peribronchiolitis with large lymphoid cuffs, bronchiectasis with purulent exudates, pulmonary atelectasis, and hyperplastic bronchial epithelia lacking visible cilia (Figure 2).

### Experiment 2

On day 21 following release of the inoculated bighorn into pen #1, *M. ovipneumoniae* was detected in the inoculated animal and one pen mate (BHS #38 and #39); the third animal (BHS #40) evaded capture and sampling on that day. The first signs of respiratory disease were observed in pen #1 animals on day 21 during drive net capture for sampling, apparently triggered by exertion (Figure 2a). On day 34, inoculated BHS #39 died in pen #1. On day 49, signs of respiratory disease were first observed in the bighorn sheep in pen #2 (Figure 2b). On days 65 and 109, #41, and #42 in pen #2 died or were euthanized *in extremis*. The surviving three bighorn sheep exhibited varying degrees of respiratory disease: BHS #38 showed persistent respiratory disease, while BHS #40 and #C showed decreasing respiratory disease over time, which became minimal after days 161 and 154, respectively. On day 204, the three surviving bighorn sheep were euthanized for necropsy. At necropsy, significant abnormal findings were limited to the respiratory tract. All six bighorn sheep had bronchopneumonia, with consolidation of lung tissue volumes ranging from an estimated 5% (BHS #40) to 80–100% (BHS #41) (Figure 2). Histopathological examination revealed severe peribronchiolitis with large lymphoid cuffs as seen in experiment 1. Animals that died or were euthanized in extremis had an overlying necrotizing bronchiolitis (#39) or abscessing bronchiolitis with bronchiectasis (BHS #41, #42) (Figure 2).

### Microbiology

All bighorn sheep in both experiments seroconverted to *M. ovipneumoniae* (Table 2). Most experimental animals had neutralizing antibody to parainfluenza-3 virus, but no significant changes in antibody titers were observed during the experimental period. Detectable antibody to other ovine respiratory viruses, including border disease virus, ovine progressive pneumonia virus, and respiratory syncytial virus was occasionally observed in single samples.

**Table 2:** Antibody responses to *M. ovipneumoniae* and parainfluenza-3 (PI-3) virus.

			*M. ovipneumoniae* 1		PI-3 virus^2^	
Experiment	ID	Pen	Pre^3^	Post^3^	Pre^3^	Post^3^
1	82	1	–8%	93%	512	512
1	89	2	–7%	88%	128	128
1	07	3	–1%	92%	256	512
2	38	1	–6%	74%	Neg	64
2	39	1	–13%	67%	Neg	<32
2	40	1	–23%	75%	64	512
2	41	2	–19%	82%	512	NT
2	42	2	–11%	82%	256	NT
2	C	2	–4%	66%	256	512

1 *M. ovipneumoniae* antibody detected by cELISA, expressed as percentage inhibition of the binding of an agent-specific monoclonal antibody [14], [18].

2 PI-3 virus neutralizing antibody detected by virus neutralization [37].

3 Pre samples in experiment 1 were obtained on the day that the *M. ovipneumoniae* colonized domestic sheep was introduced to pen 1 and in experiment 2 were obtained on the day that BHS #39 was inoculated with *M. ovipneumoniae*. ‘Post’ samples in both experiments were obtained at necropsy. Neg = No titer detected. NT = Not tested, due to inadequate specimen volume.

*M. ovipneumoniae* was detected at necropsy in both upper and lower respiratory tracts of all bighorn sheep except BHS #40 whose lung tissues were PCR negative and whose upper respiratory samples were PCR indeterminate (Table 3). Aerobic cultures and/or PCR tests identified *B. trehalosi* from pneumonic lung tissues from all bighorn sheep in both experiments (Table 3). *B. trehalosi* isolates from BHS #82 and #07 carried *lktA* and expressed leukotoxin activity (Table 3). *P. multocida* and *M. haemolytica* were not detected in these animals by either aerobic culture or PCR.

**Table 3:** Microbiologic findings from pneumonic lung tissues, based on aerobic culture and species specific PCR.

Expt.	ID	Bacterial pathogens identified in pneumonic lung tissues				
		*B. trehalosi*	*M. haemolytica*	*lktA*	*M. ovipneumoniae*	Other^5^
1	82	Cult, *sodA* 1	Neg^2^	Pos^3^	16S^4^	None
1	89	Cult, *sodA*	Neg	Neg^3^	16S	*Pasteurella* sp.^5^
1	07	Cult, *sodA*	Neg	Pos	16S	*Pasteurella* sp.
2	38	Cult, *sodA*	Neg	Neg	16S	*Pasteurella* sp.
2	39	NT, *sodA*	NT, Neg^2^	Neg	16S	NT^5^
2	40	Cult	Neg	Neg	Neg^4^	*Trueperella pyogenes* 5
2	41	Cult, *sodA*	Neg	Neg	16S	None
2	42	Cult	Neg	Neg	16S	None
2	C	Cult	Neg	Neg	16S	*Pasteurella* sp.

1 Cult = *B. trehalosi* detected by bacterial culture; *sodA = B. trehalosi* detected by *sodA* species-specific PCR (Table 1); NT = Unable to test by bacterial culture (overgrowth by *Proteus* sp.).

2 Neg = *M. haemolytica* not detected by either bacterial culture or by PCR with either *gcp* primer set (Table 1); NT = Unable to test by bacterial culture (overgrowth by *Proteus* sp.).

3 Neg = Pasteurellaceae *lktA* not detected in DNA extracts from pneumonic lung tissues by two different *lktA* PCRs (Table 1) [34], [36]. Pos = *lktA* detected in *B. trehalosi* isolates obtained from BHS #82 and #07 [36].

4 16S = *M. ovipneumoniae* detected by PCR (Table 1) [20]; Neg = *M. ovipneumoniae* not detected by PCR.

5 *Pasteurella* sp., *Trueperella pyogenes* = Bacteria isolated and identified by aerobic culture; *Pasteurella* sp. were determined not to be *B. trehalosi, M. haemolytica,* or *P. multocida*; NT = Unable to test by bacterial culture due to overgrowth by *Proteus* sp.

### Culture independent survey of bacteria in pneumonic bighorn sheep lung tissues

DNA sequences of cloned 16S rDNA revealed that the predominant bacterial species in pneumonic sections of lung were diverse (Table 4). In experiment 1, *M. ovipneumoniae* was detected in the lung tissues of all animals. *B. trehalosi* also comprised substantial proportions of the pneumonic lung flora in two animals (BHS #82 and #07), while obligate anaerobic species, primarily Fusobacterium spp., predominated in the third animal (BHS #89). The flora identified in the pneumonic lungs of the animals in experiment 2 was also substantially comprised of mixed obligate anaerobes especially *Fusobacterium* spp. (Table 4).

**Table 4:** Microbiologic findings by 16S clone library (culture independent) method.

Expt.	ID	Bacterial species identified in pneumonic lung tissues					
		Btre^1^	Movi^1^	Fuso^1^	Prev^1^	Porphyro^1^	Other^1^
1	82	20 (62.5)^2^	8 (25)	0	3 (9.4)	0	1 (3.1)
1	89	1 (3.1)	7 (21.9)	21 (65.6)	1 (3.1)	0	2 (6.3)
1	07	16 (50.0)	12 (37.5)	0	0	0	4 (12.5)
2	38	4 (7.1)	2 (3.6)	8 (14.3)	20 (35.7)	9 (16.1)	13 (23.2)
2	C	0	0	17 (30.4)	5 (8.9)	19 (33.9)	15 (26.8)
2	39	2 (6.3)	0	24 (75.0)	0	0	6 (18.8)
2	40	0	0	0	0	0	56 (100.0)
2	41	1 (3.1)	0	21 (65.6)	5 (15.6)	0	5 (15.6)
2	42	0	0	31 (96.9)	0	0	1 (3.1)

1 Btre = *B. trehalosi;* Movi = *M. ovipneumoniae;* Fuso = *Fusobacterium* sp.; Prev = *Prevotella* sp.; Porphyro = *Porphyromonas* sp.; Other = taxa other than those previously listed, each comprising <5% of sequenced clones.

2 N (%) of the sequenced 16S clones from each animal whose DNA sequences were identical to those of the tabulated bacterial species in each column.

#### Molecular epidemiology of respiratory pathogens

Consistent with epidemic transmission, *M. ovipneumoniae* strains recovered from all experimental sheep within each experiment shared identical IGS DNA sequences with the respective challenge inoculum (GenBank HQ615162 in experiment 1; KJ551511 in experiment 2).

## Discussion

The most striking finding of these experiments was the high transmissibility of *M. ovipneumoniae* and the consistent development of pneumonia that followed infection of bighorn sheep. The bacterium was naturally transmitted from single experimentally inoculated animals (a domestic sheep in experiment 1 and a bighorn sheep in experiment 2) to all animals within and between pens up to 12 m distant. Eight of nine bighorn sheep exposed to *M. ovipneumoniae* developed severe bronchopneumonia and three died, while all the domestic sheep remained healthy.

Previous experimental challenge studies conducted with *M. haemolytica or B. trehalosi* in the absence of *M. ovipneumoniae* have not documented transmission. For example, Foreyt et al. [8] reported a series of three experiments in which commingled bighorn sheep were either challenged with intra-tracheal *M. haemolytica* or given sterile BHI as controls. Four of the five control bighorn sheep survived without evidence of disease while commingled with eight *M. haemolytica-*challenged bighorn sheep, of which seven died of pneumonia [8]. Commingled bighorn sheep also remained healthy in several other studies where individual bighorn sheep died with apparent *M. haemolytica* bronchopneumonia (confirmed by isolation of this bacterium from lung tissues) [15], [25], [26].

In addition to high transmissibility, the time course of disease development and the predominant microbiology of the pneumonic lung tissues following experimental introduction of *M. ovipneumoniae* differed from that seen in previous bighorn sheep challenge experiments with other respiratory pathogens. Bighorn sheep directly challenged with leukotoxin positive *M. haemolytica* or *B. trehalosi* develop peracute bronchopneumonia and >90% die within a week of challenges with 10^5^ cfu or more [16], [27]–[30]. In contrast, disease following experimental *M. ovipneumoniae* exposures was considerably slower in onset (14–21 days post infection) and development (deaths occurring 34 to 109 days post infection; respiratory disease persisted up to 6 months post-infection); this slow time course closely resembles that documented previously in bighorn lamb pneumonia outbreaks [13]. After lethal *M. haemolytica* challenge, the agent is typically isolated from lung tissues in high numbers and pure cultures [15], [25]; in contrast in naturally occurring pneumonia outbreaks *M. ovipneumoniae* may be predominant early in the disease course but 16S library analyses have been used to document its overgrowth by diverse other bacteria later in the disease course [14], [23]. Although the numbers of animals in the experimental *M. ovipneumoniae* infection studies reported here are small, the results are consistent with the trend for early predominance of *M. ovipneumoniae* followed by overgrowth by diverse other bacterial later in the disease course (Tables 3 and 4) [13], [14], [23].

Our results also differ from our previous attempt to experimentally reproduce respiratory disease by challenge inoculation of 1-week-old bighorn lambs with *M. ovipneumoniae*, which produced minor lesions and seroconversion but no clinically significant respiratory disease [13]. However, laboratory passage of *M. ovipneumoniae* (as was performed in that experiment) has been reported to attenuate virulence in *M. ovipneumoniae* [31]. Challenge of bighorn sheep with un-passaged *M. ovipneumoniae* produced different results, as observed here in experiment #2. In another study [16], nasal washings from domestic sheep naturally colonized with *M. ovipneumoniae* or lung homogenates from a *M. ovipneumoniae-*infected bighorn sheep were used for challenge of bighorn sheep after ceftiofur treatment to eliminate detectable Pasteurellaceae. Consistent with increased virulence of un-passaged *M. ovipneumoniae*, infection and respiratory disease signs were observed in all four bighorn sheep, one of which died 19 days following challenge. The three surviving animals continued to exhibit respiratory disease signs for 42 days, at which time the experiment was terminated by challenge with *M. haemolytica* (using a dose documented to be rapidly fatal to bighorn sheep even in the absence of *M. ovipneumoniae*) [16]. As a result, the longer term effects of the mycoplasma infection were not determined in that study. Therefore, the experiments reported here are the first in which naïve bighorn sheep were exposed to un-passaged *M. ovipneumoniae* and then followed over a time period comparable with the naturally occurring disease course.

The possibility of viral agents contributing to the disease observed in this study cannot be completely ruled out, since the inoculum was derived from nasal washings from domestic sheep and no virucidal treatments were applied. However, a previous study using ultrafiltrates of bighorn sheep pneumonic lung tissues or nasal washings from domestic sheep failed to reproduce any respiratory disease in inoculated susceptible bighorn sheep [16]. In addition, serologic monitoring for the predominant domestic sheep respiratory viruses did not demonstrate seroconversion of the experimental animals in this study, as described in the Results and in Table 2. Therefore, the most parsimonious interpretation of the data presented here is that the disease observed resulted from *M. ovipneumoniae* infection and the sequelae of that infection.

The transmission of *M. ovipneumoniae* from pen-to-pen in these experiments strongly suggests that direct contact is not necessary for epizootic spread of pneumonia in bighorn sheep. Feeding, watering and other procedures involving animal care or research staff were designed to minimize the risk of human or fomite-mediated transmission of the pathogen from pen to pen, although we recognize it is impossible to completely rule out this possibility. On the other hand, since aerosolized droplet transmission is recognized as a transmission route for the closely related bacterium, *Mycoplasma hyopneumoniae* (the cause of atypical pneumonia of swine) [32], it is plausible that a similar transmission mode occurs with *M. ovipneumoniae*. Infectious aerosols generated by coughing animals would likely contribute to the explosive nature of the pneumonia outbreaks observed following initial introduction of *M. ovipneumoniae* into naïve bighorn sheep populations.

In conclusion, we demonstrated that experimental *M. ovipneumoniae* infection of naïve bighorn sheep induces chronic, severe bronchopneumonia associated with multiple secondary bacterial infections and that this infection spread rapidly to animals both within the same pen and to animals in nearby pens. The significance of these findings would be clarified by parallel experiments specifically designed to determine transmissibility and associated disease outcomes in other agents associated with bighorn sheep pneumonia, particularly *M. haemolytica,* in the absence of *M. ovipneumoniae.* Furthermore, the case-fatality rates of *M. ovipneumoniae* infected animals described here contrasts with the nearly 100% mortality that follows experimental commingling of bighorn sheep with presumptively or documented *M. ovipneumoniae-*positive domestic sheep and suggests an important role for polymicrobial secondary infections in determining mortality rates, which could be investigated in future studies. Finally, *M. ovipneumoniae* was still detected in nasal swab samples of several surviving bighorn sheep that were euthanized at the completion of these studies, suggesting that survivors of naturally occurring pneumonia outbreaks may continue to carry and shed this agent in nasal secretions. Such carriage may provide a mechanism for the post-invasion disease epizootics in lambs described in free-ranging populations. If so, this presumptive carrier state requires further study to characterize the factors that determine its occurrence and persistence, as these may be critical for the development of effective management control measures for this devastating disease.

## References

[1] Festa-Bianchet M (2008) *Ovis canadensis* The IUCN Red List of Threatened Species. Available: http://www.iucnredlist.org/details/summary/15735/0. Accessed 2014 Jul 24.

[2] Cassirer EF, Plowright RK, Manlove KR, Cross PC, Dobson AP, et al (2012) Spatio-temporal dynamics of pneumonia in bighorn sheep (*Ovis canadensis*). J Animal Ecol 82: 518–528. 10.1111/1365-2656.12031 23398603

[3] Marsh H (1938) Pneumonia in Rocky Mountain bighorn sheep. J Mammal 19: 214–219.

[4] Buechner HK (1960) The bighorn sheep in the United States, its past, present, and future. Wildl Monog 4: 3–174.

[5] Forrester DJ (1971) Bighorn sheep lungworm-pneumonia complex. In: Davis JW, Anderson RC, editors. Parasitic Diseases of Wild Mammals. Ames, IA: Iowa State University Press. 158–173.

[6] Demartini JC, Davies RB (1977) An epizootic of pneumonia in captive bighorn sheep infected with *Muellerius* sp. J Wildl Dis 13: 117–124. 864843 10.7589/0090-3558-13.2.117

[7] Miller MW (2001) Pasteurellosis. In: Williams ES, Barker, I K., editor. Infectious diseases of wild mammals. Ames IA USA: Iowa State University Press. 558.

[8] Foreyt WJ, Snipes KP, Kasten RW (1994) Fatal pneumonia following inoculation of healthy bighorn sheep with *Pasteurella haemolytica* from healthy domestic sheep. J Wildl Dis 30: 137–145. 8028096 10.7589/0090-3558-30.2.137

[9] Kraabel BJ, Miller MW, Conlon JA, McNeil HJ (1998) Evaluation of a multivalent *Pasteurella haemolytica* vaccine in bighorn sheep: Protection from experimental challenge. J Wildl Dis 34: 325–333. 9577780 10.7589/0090-3558-34.2.325

[10] Rudolph KM, Hunter DL, Foreyt WJ, Cassirer EF, Rimler RB, et al (2003) Sharing of *Pasteurella* spp. between free-ranging bighorn sheep and feral goats. J Wildl Dis 39: 897–903. 14733287 10.7589/0090-3558-39.4.897

[11] Rudolph KM, Hunter DL, Rimler RB, Cassirer EF, Foreyt WJ, et al (2007) Microorganisms associated with a pneumonic epizootic in Rocky Mountain bighorn sheep (*Ovis canadensis canadensis*). J Zoo Wildl Med 38: 548–558. 18229860 10.1638/2006-0027R.1

[12] Weiser GC, DeLong WJ, Paz JL, Shafii B, Price WJ, et al (2003) Characterization of *Pasteurella multocida* associated with pneumonia in bighorn sheep. J Wildl Dis 39: 536–544. 14567214 10.7589/0090-3558-39.3.536

[13] Besser TE, Cassirer EF, Potter KA, VanderSchalie J, Fischer A, et al (2008) Association of *Mycoplasma ovipneumoniae* infection with population-limiting respiratory disease in free-ranging rocky mountain bighorn sheep (*Ovis canadensis canadensis*). J Clin Microbiol 46: 423–430. 18057131 10.1128/JCM.01931-07 PMC2238132

[14] Besser TE, EF Cassirer, MA Highland, P Wolff, A Justice-Allen (2012) et.al (2012) Bighorn sheep pneumonia: Sorting out the cause of a polymicrobial disease. Prev Vet Med 108: 85–93. 23253148 10.1016/j.prevetmed.2012.11.018

[15] Besser TE, Cassirer EF, Yamada C, Potter KA, Herndon C, et al (2012) Survival of Bighorn Sheep (*Ovis canadensis*) Commingled with Domestic Sheep (*Ovis aries*) in the Absence of *Mycoplasma ovipneumoniae* . J Wildl Dis 48: 168–172. 22247385 10.7589/0090-3558-48.1.168

[16] Dassanayake RP, Shanthalingam S, Herndon CN, Subramaniam R, Lawrence PK, et al (2010) *Mycoplasma ovipneumoniae* can predispose bighorn sheep to fatal *Mannheimia haemolytica* pneumonia. Vet Microbiol 145: 354–359. 20466492 10.1016/j.vetmic.2010.04.011

[17] Blodgett R (2010) Bacteriologic Analytical Manual Appendix 2: Most Probable Number from Serial Dilutions. Washington DC. Available: http://www.fda.gov/Food/FoodScience Research/LaboratoryMethods/ucm109656.htm. Accessed 2014 Jul 24.

[18] Ziegler JC, Lahmers KK, Barrington GM, Parish SM, Kilzer K, et al (2014) Safety and Immunogenicity of a Mycoplasma ovipneumoniae Bacterin for Domestic Sheep (*Ovis aries*). PLoS One 9(4): e95698. 24752006 10.1371/journal.pone.0095698 PMC3994082

[19] Herrmann LM, Cheevers WP, Marshall KL, McGuire TC, Hutton MM, et al (2003) Detection of serum antibodies to ovine progressive pneumonia virus in sheep by using a caprine arthritis-encephalitis virus competitive-inhibition enzyme-linked immunosorbent assay. Clin Diag Lab Immunol 10: 862–865. 10.1128/CDLI.10.5.862-865.2003 PMC193903 12965917

[20] Lawrence PK, Shanthalingam S, Dassanayake RP, Subramaniam R, Herndon CN, et al (2010) Transmission of *Mannheimia haemolytica* from domestic sheep (*Ovis aries*) to bighorn sheep (*Ovis canadensis*): unequivocal demonstration with green fluorescent protein-tagged organisms. J Wildl Dis 46: 706–717; erratum, J Wildl Dis 46: 1346. 10.7589/0090-3558-46.3.706 20688676

[21] McAuliffe L, Hatchell FM, Ayling RD, King AI, Nicholas RA (2003) Detection of *Mycoplasma ovipneumoniae* in *Pasteurella*-vaccinated sheep flocks with respiratory disease in England. Vet Rec 153: 687–688. 14682543 10.1136/vr.153.22.687

[22] Gentry MJ, Srikumaran S (1991) Neutralizing monoclonal antibodies to *Pasteurella haemolytica l*eukotoxin affinity purify the toxin from crude culture supernatants. Microb Pathog 10: 411–417. 1753879 10.1016/0882-4010(91)90086-p

[23] Besser TE, Highland M, Baker K, Anderson NJ, Ramsey JM (2012) Causes of pneumonia epizootics among bighorn sheep, western United States, 2008–2010. Emerg Infect Dis 18: 406–414. 22377321 10.3201/eid1803.111554 PMC3309594

[24] Petti CA (2007) Detection and identification of microorganisms by gene amplification and sequencing. Clin Infect Dis 44: 1108–1114. 17366460 10.1086/512818

[25] Foreyt WJ, Jenkins EJ, Appleyard GD (2009) Transmission of lungworms (*Muellerius capillaris*) from domestic goats to bighorn sheep on common pasture. J Wildl Dis 45: 272–278. 19395736 10.7589/0090-3558-45.2.272

[26] Foreyt WJ, Lagerquist JE (1996) Experimental contact of bighorn sheep (*Ovis canadensis*) with horses and cattle, and comparison of neutrophil sensitivity to *Pasteurella haemolytica* cytotoxins. J Wildl Dis 32: 594–602. 9359057 10.7589/0090-3558-32.4.594

[27] Onderka DK, Rawluk SA, Wishart WD (1988) Susceptibility of Rocky Mountain bighorn sheep and domestic sheep to pneumonia induced by bighorn and domestic livestock strains of *Pasteurella haemolytica* . Can J Vet Res 52: 439–444. 3196974 PMC1255488

[28] Dassanayake RP, Shanthalingam S, Herndon CN, Lawrence PK, Cassirer EF, et al (2009) *Mannheimia haemolytica* serotype A1 exhibits differential pathogenicity in two related species, *Ovis canadensis* and *Ovis aries* . Vet Microbiol 133: 366–371. 18771862 10.1016/j.vetmic.2008.07.015

[29] Subramaniam R, Shanthalingam S, Bavananthasivam J, Kugadas A, Potter KA, et al (2011) A multivalent *Mannheimia-Bibersteinia* vaccine protects bighorn sheep against *Mannheimia haemolytica* challenge. Clin Vaccine Immunol 18: 1689–1694. 21832104 10.1128/CVI.05276-11 PMC3187036

[30] Foreyt WJ, Snipes KP, Kasten RW (1994) Fatal pneumonia following inoculation of healthy bighorn sheep with *Pasteurella haemolytica* from healthy domestic sheep. J Wildl Dis 30: 137–145. 8028096 10.7589/0090-3558-30.2.137

[31] Alley MR, Ionas G, Clarke JK (1999) Chronic non-progressive pneumonia of sheep in New Zealand - a review of the role of *Mycoplasma ovipneumoniae* . N Z Vet J 47: 155–160. 16032095 10.1080/00480169.1999.36135

[32] Desrosiers R (2011) Transmission of swine pathogens: different means, different needs. Anim Health Res Rev 12: 1–13. 21241537 10.1017/S1466252310000204

[33] Dassanayake RP, Call DR, Sawant AA, Casavant NC, Weiser GC, et al (2010) *Bibersteinia trehalosi* inhibits the growth of *Mannheimia haemolytica* by a proximity-dependent mechanism. Appl Environ Microbiol 76: 1008–1013. 20038698 10.1128/AEM.02086-09 PMC2820941

[34] Shanthalingam S, Goldy A, Bavananthasivam J, Subramaniam R, Batra SA, et al (2014) PCR assay detects *Mannheimia haemolytica i*n culture-negative pneumonic lung tissues of bighorn sheep (*Ovis canadensis*) from outbreaks in the Western USA, 2009–2010. J Wildl Dis 50: 1–10. 24171569 10.7589/2012-09-225

[35] Townsend KM, Frost AJ, Lee CW, Papadimitriou JM, Dawkins HJ (1998) Development of PCR assays for species- and type-specific identification of *Pasteurella multocida* isolates. J Clin Microbiol 36: 1096–1100. 9542944 10.1128/jcm.36.4.1096-1100.1998 PMC104696

[36] Fisher MA, Weiser GC, Hunter DL, Ward ACS (1999) Use of a polymerase chain reaction method to detect the leukotoxin gene IktA in biogroup and biovariant isolates of *Pasteurella haemolytica* and *P. trehalosi* . Am J Vet Res 60: 1402–1406. 10566816

[37] Rossi CR, Kiesel GK (1971) Microtiter tests for detecting antibody in bovine serum to parainfluenza 3 virus, infectious bovine rhinotracheitis virus, and bovine virus diarrhea virus. Appl Microbiol 22: 32–36. 4329432 10.1128/am.22.1.32-36.1971 PMC377372

